# Tumor Necrosis Factor B (*TNFB*) Genetic Variants and Its Increased Expression Are Associated with Vitiligo Susceptibility

**DOI:** 10.1371/journal.pone.0081736

**Published:** 2013-11-27

**Authors:** Naresh C. Laddha, Mitesh Dwivedi, Amina R. Gani, Mohmmad Shoab Mansuri, Rasheedunnisa Begum

**Affiliations:** Department of Biochemistry, Faculty of Science, The Maharaja Sayajirao University of Baroda, Vadodara, India; South Texas Veterans Health Care System and University of Texas Health Science Center at San Antonio, United States of America

## Abstract

Genetic polymorphisms in *TNFB* are involved in the regulation of its expression and are found to be associated with various autoimmune diseases. The aim of the present study was to determine whether *TNFB* +252A/G (rs909253) and exon 3 C/A (rs1041981) polymorphisms are associated with vitiligo susceptibility, and expression of *TNFB* and *ICAM1* affects the disease onset and progression. We have earlier reported the role of *TNFA* in autoimmune pathogenesis of vitiligo, and we now show the involvement of *TNFB* in vitiligo pathogenesis. The two polymorphisms investigated in the *TNFB* were in strong linkage disequilibrium and significantly associated with vitiligo. *TNFB* and *ICAM1* transcripts were significantly increased in patients compared to controls. Active vitiligo patients showed significant increase in *TNFB* transcripts compared to stable vitiligo. The genotype-phenotype analysis revealed that *TNFB* expression levels were higher in patients with GG and AA genotypes as compared to controls. Patients with the early age of onset and female patients showed higher *TNFB* and *ICAM1* expression. Overall, our findings suggest that the increased *TNFB* transcript levels in vitiligo patients could result, at least in part, from variations at the genetic level which in turn leads to increased *ICAM1* expression. For the first time, we show that *TNFB* +252A/G and exon 3 C/A polymorphisms are associated with vitiligo susceptibility and influence the *TNFB* and *ICAM1* expression. Moreover, the study also emphasizes influence of *TNFB* and *ICAM1* on the disease progression, onset and gender bias for developing vitiligo.

## Introduction

Vitiligo is an acquired, hypomelanotic disease characterized by circumscribed depigmented macules. The absence of melanocytes from the lesional skin due to their destruction has been suggested to be the key event in the pathogenesis of vitiligo [1]. It is a polygenic, multifactorial disorder involving multiple susceptibility genes and unknown environmental triggers [2]–[4]. It affects approximately 0.5–1% of the world population [5]. The exact etiology of vitiligo remains obscure, but autoimmunity has been strongly implicated in the development of disease as approximately 30% of vitiligo patients are affected with at least one additional autoimmune disorder [6], [7]. Epidemiological studies have shown frequent family clustering of vitiligo cases, with elevated risk of vitiligo in first-degree relatives and high concordance in monozygotic twins [4], [6], suggesting a genetic basis for vitiligo. A number of genes have been implicated in the pathogenesis of vitiligo on the basis of genetic linkage and association studies, including *MHC*, *ACE*, *CAT*, *CTLA4*, *COMT*, *ESR*, *MBL2*, *PTPN22, HLA*, *NALP1, XBP1, FOXP1* and *IL2RA* that are involved in the regulation of immunity [8], [9].

Cytokines are important mediators of immunity and there is now convincing evidence that cytokines also have an important role in the pathogenesis of autoimmunity [10]. Morreti et al. [11], [12] have shown cytokine imbalance in the skin of vitiligo patients suggesting their role in autoimmune pathogenesis of vitiligo. Analysis of cytokine gene polymorphisms identifies genetic abnormalities of cytokine regulation, thus allows us to establish genotype-phenotype correlation which may be important in unraveling the disease pathogenesis.

Tumor necrosis factor B (TNFB) also known as lymphotoxin A (LTA), is a close homologue of TNFA. Both these cytokines are recognized by the same widely distributed cellular TNF receptors i.e. TNFR1 and TNFR2, as a consequence, many of their effects are similar [13]. TNFB is a Th1 cytokine, primarily produced by activated T and B lymphocytes. It is a potent mediator of inflammatory and immune responses and it is also involved in the regulation of various biological processes including cell proliferation, differentiation, apoptosis, lipid metabolism, coagulation and neurotransmission [14]. The *TNFB* gene contains four exons and encodes a primary transcript of 2038 nucleotides. TNFB is a protein of 171 amino acids and secreted as a soluble polypeptide, but can form heterotrimers with lymphotoxin-beta, which effectively anchors the TNFB to the cell surface [15]. TNFB induces cell apoptosis upon binding to TNFR1, but it induces inflammatory responses by activating NF-kB nuclear protein upon binding to TNFR2 [16].

Polymorphisms of *TNFA* and *TNFB* genes have been shown to affect their production and hence can be associated with several autoimmune diseases [14], [17]. We have earlier reported the crucial role of *TNFA* in autoimmune pathogenesis of vitiligo [18], and we now show the involvement of *TNFB* and its downstream effector, intercellular adhesion molecule 1 *(ICAM1)* in vitiligo pathogenesis. Two well-characterized variants of *TNFB* are in tight linkage disequilibrium [intron 1 +252A/G (rs909253; IVS1+90 A/G) and exon 3 C/A (rs1041981; Thr26Asn)] are found to influence *TNFB* expression *in vitro* [19], [20]. The +252A/G polymorphism was reported to influence TNFB plasma levels. This single nucleotide polymorphism (A/G) affects a phorbol ester-response element and distinguishes the two alleles that have been designated as *TNFB1* and *TNFB2* [19]. *TNFA2* and *TNFB2* alleles have much more powerful transcriptional activator binding sites than *TNFA1* and *TNFB1* alleles, respectively [19]. Other *in vitro* experiments have shown that *TNFB* exon 3 C/A (Thr26Asn) polymorphism is associated with a two fold increase in the induction of several cell-adhesion molecules including ICAM1 in the smooth muscle cells of human coronary arteries [21].

It has been suggested that melanocyte death is mediated by apoptosis in the context of autoimmunity, and cytokines such as IFNG, TNFA, TNFB and IL1 can initiate apoptosis [22]. Additionally, IFNG, TNFA and TNFB induce the expression of ICAM1 on the cell-surface of melanocytes [23]. Increased expression of this adhesion molecule on the melanocytes enhances T cell- melanocyte attachment in the skin and may play a role in the destruction of melanocytes in vitiligo [24]. These reports signify that the host's ability to produce cytokines such as TNFA and TNFB may play a crucial role in vitiligo susceptibility.

In the present study, we have made an attempt to understand the role of *TNFB* and *ICAM1* in vitiligo pathogenesis. The objectives of this study were: i.) to determine whether the single nucleotide polymorphisms (SNPs) of *TNFB* [intron 1 +252A/G (rs909253; IVS1+90 A/G) and exon 3 C/A (rs1041981; Thr26Asn)] are associated with vitiligo susceptibility; ii.) to estimate and compare *TNFB* and *ICAM1* transcript levels in patients and unaffected controls; iii.) to correlate *TNFB* and *ICAM1* transcript levels with onset and progression of the disease.

## Materials and Methods

### Study subjects

The study group included 524 vitiligo patients comprised of 224 males and 300 females who referred to S.S.G. Hospital, Vadodara and Civil Hospital, Ahmedabad, Gujarat, India (Table 1).

**Table 1:** Demographic characteristics of vitiligo patients and unaffected controls.

Characteristic	Vitiligo Patients	Controls
	n = 524	n = 592
Average age(mean age ± SD), yrs	31.24 ± 12.13	27.54 ± 13.26
Sex: Male, No. (%)Female, No. (%)	224 (42.75)300 (57.25)	267 (45.10)325 (54.90)
Age of onset(mean age ± SD), yrsDuration of disease(mean ± SD), yrs	21.96 ± 14.908.20 ± 7.11	NANA
Type of vitiligo:		
Generalized, No. (%)Localized, No. (%)Active, No. (%)Stable, No. (%)Family history, No. (%)	360 (68.70)164 (31.30)393 (75.00)131 (25.00)68 (12.98)	NANANANANA

‘n’ represents number of patients/controls, SD refers to standard deviation, NA refers to not applicable, yrs refers to years, No. refers to numbers.

The diagnosis of vitiligo was clinically based on characteristic skin depigmentation with typical localization and white colour on the skin lesions under Woods lamp and confirmed as two different types: generalized (including acrofacial vitiligo and vitiligo universalis) and localized by dermatologists. Patients had no other associated autoimmune disease. The patients were divided into two subgroups: active vitiligo (existing lesions were spreading and/or new lesions had appeared within the previous 6 months) and stable vitiligo (no increase in lesion size or number within 6 months or more). Three hundred and ninety three patients were classified with active vitiligo, whereas 131 patients were included in the stable vitiligo group (Table 1).

A total of 592 ethnically age- and sex-matched unaffected individuals (267 males and 325 females) were included in the study (Table 1). None of the healthy individuals or their relatives had any evidence of vitiligo and autoimmune disease. The study plan was approved by the Institutional Ethics Committee for Human Research (IECHR), Faculty of Science, The Maharaja Sayajirao University of Baroda, Vadodara, Gujarat, India. The importance of the study was explained to all participants and written consent was obtained from all subjects before performing the studies. The written consent was also obtained from the guardians on behalf of the children participants involved in the study. IECHR approved the written consent procedure for patient and control participants.

### Genotyping of *TNFB* +252A/G polymorphism

Polymerase chain reaction-restriction fragment length polymorphism (PCR-RFLP) was used to genotype *TNFB* +252A/G polymorphism. Five ml venous blood was collected from the patients and healthy subjects in K_3_EDTA coated vacutainers (BD, Franklin Lakes, NJ 07417, USA). Genomic DNA was extracted from whole blood using ‘QIAamp DNA Blood Kit’ (QIAGEN Inc., Valencia, CA 91355, USA) according to the manufacturer's instructions. The primers used for polymerase chain reaction are mentioned in Table S1 in File S1. The reaction mixture of the total volume of 20 µL included 5 µL (100 ng) of genomic DNA, 10 µL nuclease-free H_2_O, 2.0 µL 10x PCR buffer, 2 µL 2 mM dNTPs (SIGMA Chemical Co, St. Louis, Missouri, USA), 0.3 µL of 10 µM corresponding forward and reverse primers (Eurofins, Bangalore, India), and 0.3 µL (5 U/µL) Taq Polymerase (Bangalore Genei, Bangalore, India). Amplification was performed using a PTC-100 thermal cycler (MJ Research, Inc., Watertown, Massachusetts, USA) according to the protocol: 95°C for 10 minutes followed by 35 cycles of 95°C for 30 seconds, 62°C for 30 seconds, and 72°C for 30 seconds. The amplified products were checked by electrophoresis on a 2.0% agarose gel stained with ethidium bromide.

Restriction enzyme *Nco*I (New England Biolabs, Beverly, MA) was used for digesting amplicons of *TNFB* +252 A/G (Table S1 in File S1). 5 µL of the amplified products were digested with 5 U of the restriction enzyme in a total reaction volume of 25 µL as per the manufacturer's instruction. The digested products with 100 base pair DNA ladder (Bioron, Ludwigshafen am Rhein, Germany) were resolved on 2.0% agarose gels stained with ethidium bromide and visualized under UV transilluminator. A 417 bp undigested product corresponding to A allele and 284 bp and 133 bp digested products corresponding to G allele were identified and three different genotypes were classified as: GG homozygous, AG heterozygous and AA homozygous (Fig. S1 in File S2). More than 10% of the samples were randomly selected for confirmation and the results were 100% concordant (analysis of the chosen samples was repeated by two researchers independently).

### Genotyping of *TNFB* Thr26Asn C/A polymorphism

The genotyping of Thr26Asn C/A SNP of *TNFB* was carried out by dual colour hydrolysis probes labeled with FAM (for ‘A’ allele) and VIC (for ‘C’ allele) using LightCycler® 480Real-Time PCR protocol with background corrected end point fluorescence analysis in a TaqMan SNP genotyping assay (Assay ID: C_7514870_20; Life Technologies Corp., California, USA) which yielded the three genotypes (AA homozygous, CA heterozygous and CC homozygous) as identified on scattered plot (Fig. S2 in File S2). Real-time PCR was performed in 10 µl volume using LightCycler®480 Probes Master (Roche Diagnostics GmbH, Mannheim, Germany) following the manufacturer's instructions. A no-template control (NTC) was used with the SNP genotyping assay.

### Determination of *TNFB*, *ICAM1* and *GAPDH* mRNA expression

*TNFB, ICAM1* and *GAPDH* mRNA levels were measured in whole blood of 166 patients and 175 controls by Real-time PCR.

#### RNA extraction and cDNA synthesis

Total RNA from whole blood was isolated and purified using the RibopureTM - blood Kit (Ambion inc. Texas, USA) following the manufacturer's protocol. RNA integrity was verified by 1.5% agarose gel electrophoresis, RNA yield and purity was determined spectrophotometrically at 260/280 nm. RNA was treated with DNase I (Ambion inc. Texas, USA) before cDNA synthesis to avoid DNA contamination. cDNA synthesis was performed using 1 µg of total RNA by RevertAid First Strand cDNA Synthesis Kit (Fermentase, Vilnius, Lithuania) according to the manufacturer's instructions in the MJ Research Thermal Cycler (Model PTC-200, Watertown, MA, USA).

#### Real-time PCR

The expression of *TNFB, ICAM1* and *GAPDH* transcripts were measured by real-time PCR using gene specific primers (Eurofins, Bangalore, India) as shown in Table S1 in File S1. Expression of the *GAPDH* gene was used as a reference. Real-time PCR was performed in duplicates in 20 µl volume using LightCycler®480 SYBR Green I Master (Roche Diagnostics GmbH, Mannheim, Germany) following the manufacturer's instructions. The thermal cycling conditions included an initial activation step at 95°C for 10 min, followed by 45 cycles of denaturation, annealing and amplification (95°C for 10 sec, 65°C for 15 sec, 72°C for 20 sec.). The fluorescence data collection was performed during the extension step. At the end of the amplification phase a melt curve analysis was carried out to check the specificity of the products formed (Fig. S3 in File S2). The PCR cycle at which PCR amplification begins its exponential phase and product fluorescence intensity finally rises above the background and becomes visible was considered as the crossing point (C_P_) or cycle threshold (C_T_). The ΔC_T_ or ΔC_P_ value was determined as the difference between the cycle threshold of target genes (*TNFB/ICAM1*) and reference gene (*GAPDH*). The difference between the two ΔC_P_ values (ΔC_P_ Controls and ΔC_P_ patients) was considered as ΔΔC_P_ to obtain the value of fold expression (2^−ΔΔCp^).

### Statistical analyses

Evaluation of the Hardy-Weinberg equilibrium (HWE) was performed for both the polymorphisms in patients and controls by comparing the observed and expected frequencies of the genotypes using chi-square analysis. The distribution of the genotypes and allele frequencies of *TNFB* polymorphisms in patients and control subjects were compared by chi-square test. *p*-values less than 0.025 were considered as statistically significant due to Bonferroni's correction for multiple testing. Odds ratio (OR) with respective confidence interval (95% CI) for disease susceptibility was also calculated. Age of onset analysis and relative expression of *TNFB* and *ICAM1* in patient and control groups were plotted and analyzed by Mann-Whitney Wilcoxon test. Chi-square and Mann-Whitney Wilcoxon tests were performed using Prism 4 software (Graphpad software Inc; San Diego CA, USA, 2003). The statistical power of detection of the association with the disease at the 0.05 level of significance was determined by using the G* Power software [25].

## Results

### Analysis of association between *TNFB* +252A/G and exon 3 C/A (Thr26Asn) polymorphisms and susceptibility to vitiligo

The genotype and allele frequencies of the +252 A/G polymorphism in 524 vitiligo patients and 592 controls are summarized in Table 2. The *TNFB* +252 A/G polymorphism was found to be in significant association with vitiligo patients compared to controls (*p* = 0.002; Table 2). The minor allele (G) of +252 A/G was more frequent in the vitiligo group compared to the control group (27.0% versus 21.0%, *p* = 0.001; OR: 1.424, 95% CI: 1.171–1.732) consistent with a susceptibility effect (Table 2). The distribution of *TNFB* +252 A/G genotype frequencies was consistent with Hardy-Weinberg expectations in control and patient groups (*p*>0.05) (Table 2). Moreover, generalized vitiligo (GV) group showed significant association of +252 A/G polymorphism when the genotypes were compared with those of the control group (*p*<0.0001); however localized vitiligo (LV) group did not show significant association of the polymorphism (*p* = 0.826) (Table 3). Also, there was significant difference in allele frequencies of the polymorphism for GV group when the alleles were compared with those of the control group (*p*<0.0001); however, the LV group did not show significant difference in allele frequencies (*p* = 0.759) (Table 3). In addition, gender based analysis of *TNFB* +252 A/G polymorphism suggested significant association of minor +252 G allele with female vitligo patients as compared to male patients (30.0% versus 23.0%, *p* = 0.021); however, genotype frequencies could not achieve significance due to Bonferroni's correction for multiple testing (*p* = 0.039) (Table S2 in File S1). Similar results were obtained from the association study of *TNFB* C/A SNP (Tables S3 & S4 in File S1) as the two SNPs studied were in perfect LD (linkage disequilibrium) in our population and provide the same genetic information for the associations, and so as the haplotype. This study has 84.96% statistical power for the effect size 0.08 to detect the association of investigated polymorphisms at *p*<0.05 in vitiligo patients and control population.

**Table 2:** Association studies for *TNFB* gene +252 A/G polymorphism in vitiligo patients and controls from Gujarat.

SNP	Genotype or allele	Vitiligo Patients(Freq.)	Controls(Freq.)	*p* for Association	*p* for HWE	Odds ratio(95% CI)
rs909253(+252 A/G)	GenotypeAAAGGG	n = 524285 0.54)193 (0.37)46 (0.09)	n = 592375 (0.63)188 (0.32)29 (0.05)	0.002^a^	0.109(Patients)	
	AlleleAG	763 (0.73)285 (0.27)	938 (0.79)246 (0.21)	0.001^b^	0.389(Controls)	1.424 (1.171–1.732)

‘n’ represents number of patients/controls, HWE refers to Hardy-Weinberg Equilibrium, CI refers to confidence interval,

a vitiligo patients vs. controls using chi-square test with 3×2 contingency table,

b vitiligo patients vs. controls using chi-square test with 2×2 contingency table, values are significant at *p*≤0.025 due to Bonferroni's correction.

**Table 3:** Association studies for *TNFB* gene +252 A/G polymorphism in different clinical types of vitiligo patients and controls from Gujarat.

SNP	Genotype or allele	Generalized Vitiligo Patients(Freq.)	LocalizedVitiligo Patients(Freq.)	Controls(Freq.)	*p* for Association	*p* for HWE	Odds ratio(95% CI)
rs909253(+252 A/G)	GenotypeAAAGGGAlleleAG	n = 360184 (0.51)137 (0.38)39 (0.11)505 (0.70)215 (0.30)	n = 164103 (0.63)51 (0.31)10 (0.06)257 (0.78)71 (0.22)	n = 592375(0.63)188(0.32)29 (0.05)938(0.79)246(0.21)	0.030^a^<0.0001^b^0.826^c^0.006^a^<0.0001^b^0.759^c^	0.083(GV)0.286(LV)0.389(Controls)	1.541^a^ (1.133–2.096)1.623^b^ (1.312–2.008)1.053^c^ (0.782–1.419)
rs909253(+252 A/G)	GenotypeAAAGGGAlleleAG	ActiveVitiligo(Freq.)n = 393201 (0.51)152 (0.39)40 (0.10)554 (0.70)232 (0.30)	StableVitiligo(Freq.)n = 13185 (0.65)40 (0.30)6 (0.05)210 (0.80)52 (0.20)	Controls(Freq.)n = 592375 (0.63)188 (0.32)29 (0.05)938(0.79)246(0.21)	0.012^d^<0.0001^e^0.945^f^0.002^d^<0.0001^e^0.800^f^	0.163(AV)0.645(SV)0.389(Controls)	1.691^d^ (1.204–2.376)1.597^e^ (1.297–1.966)0.944^f^ (0.676–1.319)

‘n’ represents number of patients/controls, HWE refers to Hardy-Weinberg Equilibrium, CI refers to confidence interval, GV refers to generalized vitiligo, LV refers to localized vitiligo, AV refers to active vitiligo, SV refers to stable vitiligo,

a generalized vitiligo vs. localized vitiligo,

b generalized vitiligo vs. controls,

c localized vitiligo vs. controls,

d active vitiligo vs. stable vitiligo,

e active vitiligo vs. controls,

f stable vitiligo vs. controls, values are significant at *p*≤0.025 due to Bonferroni's correction.

### Effect of *TNFB* +252 A/G genotypes on progression of vitiligo

Analysis based on the stage of progression of vitiligo revealed that the increased frequency of the +252 G allele occurred prevalently in the group of patients with active vitiligo compared to the control group (30.00% versus 21.00%, *p*<0.0001) (Table 3). However, there was no statistically significant difference in the distribution of the +252 G allele between patients with stable vitiligo and control group (20.00% versus 21.00%, *p* = 0.800) (Table 3). Interestingly, the +252 G allele was more prevalent in patients with active vitiligo as compared to stable vitiligo (30.00% versus 20.00%, *p* = 0.002) suggesting the important role of +252 G allele in the progression of the disease (Table 3).

### Relative gene expression of *TNFB* in vitiligo patients and controls

**Figure 1:**
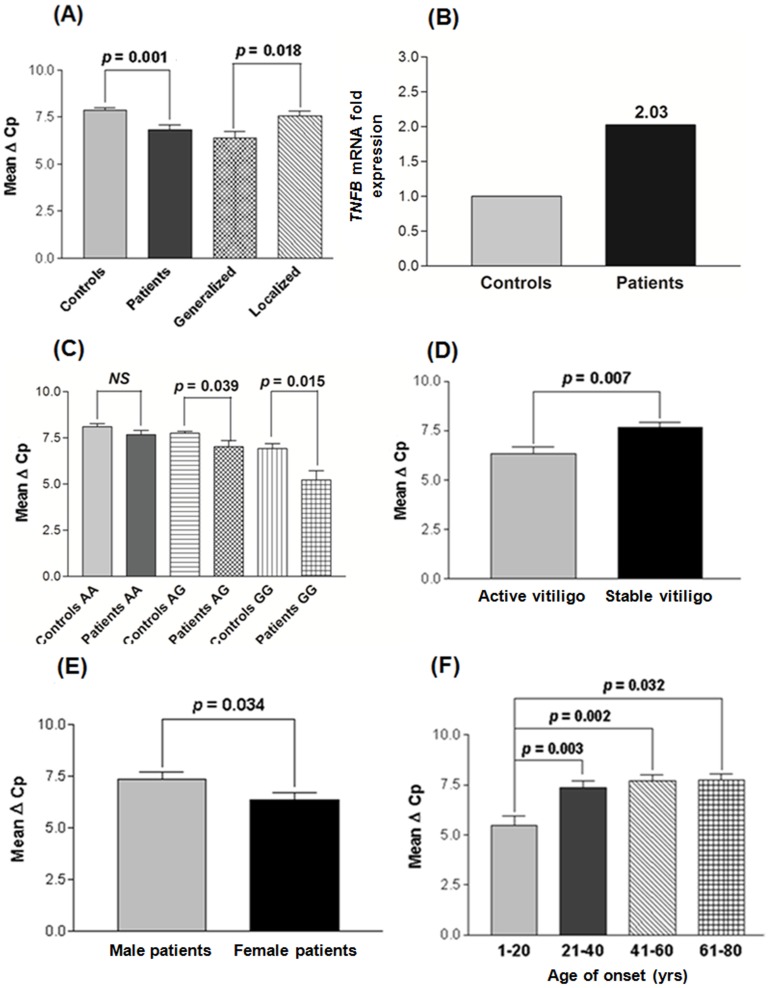
Relative gene expression of *TNFB* in controls and vitiligo patients. **(A)** Expression of *TNFB* mRNA in 175 controls, 166 vitiligo patients, 122 generalized vitiligo patients and 44 localized vitiligo patients using Mann-Whitney Wilcoxon test. **(B)** Fold expression of *TNFB* mRNA in 166 vitiligo patients and 175 controls, as determined by 2^−ΔΔCp^ method. **(C)** Expression of *TNFB* mRNA with respect to *TNFB* +252 A/G in 166 vitiligo patients and 175 controls using Mann-Whitney Wilcoxon test. **(D)** Expression of *TNFB* mRNA with respect to activity of the disease in 121 active vitiligo and 45 stable vitiligo patients using Mann-Whitney Wilcoxon test. **(E)** Expression of *TNFB* mRNA with respect to gender differences in 87 male and 79 female vitiligo patients using Mann-Whitney Wilcoxon test. **(F)** Expression of *TNFB* mRNA with respect to different age of onset groups in 166 vitiligo patients using Mann-Whitney Wilcoxon test.

Comparison of the findings showed significant increase in expression of *TNFB* mRNA in 166 vitiligo patients compared to 175 unaffected controls after normalization with *GAPDH* expression as suggested by mean ΔCp values (*p* = 0.001) (Fig. 1A). Moreover, GV patients showed significantly higher expression of *TNFB* mRNA as compared to LV patients (*p* = 0.018) (Fig. 1A). The 2^−ΔΔCp^ analysis showed approximately 2.0 fold higher expression of *TNFB* mRNA in patients as compared to controls (Fig. 1B).

### Correlation of *TNFB* transcripts with the *TNFB* +252 A/G and exon 3 C/A genotypes

Further, the expression levels of *TNFB* were analyzed with respect to +252 A/G genotypes (Fig. 1C). Interestingly, *TNFB* expression was significantly increased in patients with susceptible GG genotypes as compared to controls (*p* = 0.015). Also, patients with genotypes AG showed significant increase in *TNFB* mRNA as compared to controls (*p* = 0.039); however, no significant difference was observed in *TNFB* expression in patients as compared to controls with AA genotypes (*p* = 0.168) (Fig. 1C). As +252 A/G SNP is strongly linked with the exon 3 C/A SNP, similar results were obtained for *TNFB* expression with respect to exon 3 C/A genotypes (Fig. S4A in File S2).

### Effect of *TNFB* expression on disease progression

In addition, we also checked the effect of *TNFB* expression on progression of the disease i.e. active and stable cases of vitiligo (Fig. 1D). Interestingly, active vitiligo patients showed significant increase in expression of *TNFB* mRNA as compared to the patients with stable vitiligo (*p* = 0.007) suggesting the involvement of *TNFB* in disease progression. Moreover, the susceptibility of the disease was also checked based on the gender differences and we found that female patients with vitiligo showed significantly higher *TNFB* expression as compared to male patients (*p* = 0.034) (Fig. 1E).

When *TNFB* expression was monitored in different patient groups of age of onset, patients with the age group 1–20 yrs showed significant increase in expression of *TNFB* mRNA as compared to the age groups 21–40, 41–60 and 61–80 yrs (*p* = 0.003, *p* = 0.002 and *p* = 0.032 respectively) suggesting the importance of *TNFB* in early onset of the disease (Fig. 1F).

### Relative gene expression of *ICAM1* in patients with vitiligo and controls

**Figure 2:**
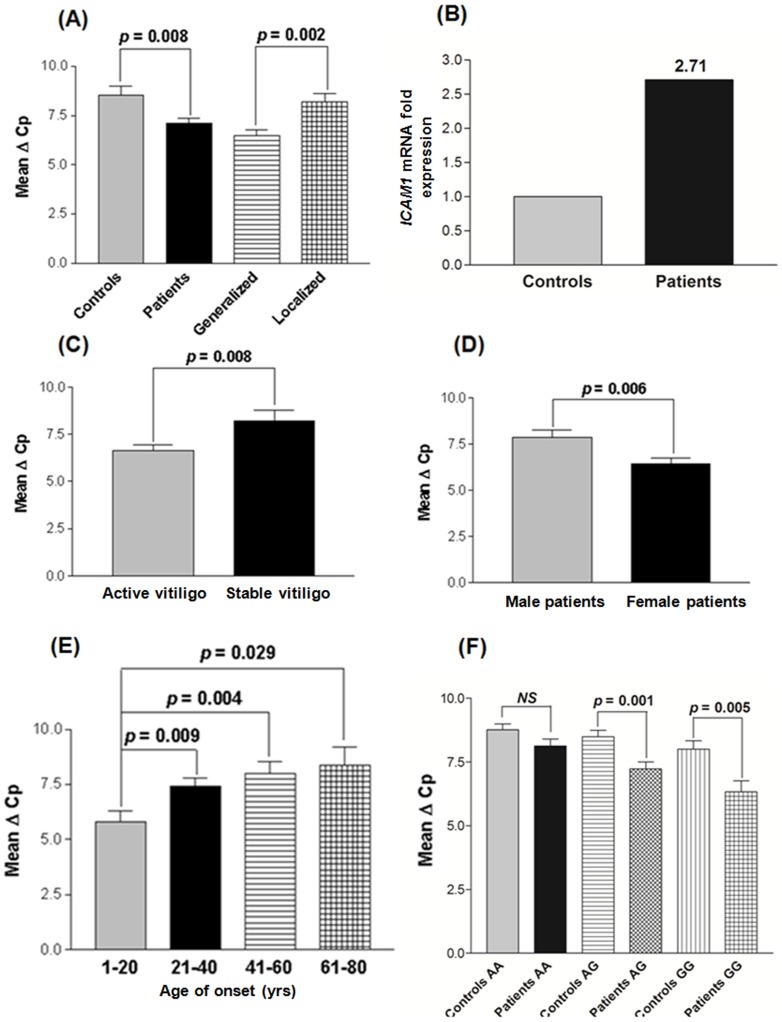
Relative gene expression of *ICAM1* in controls and vitiligo patients. **(A)** Expression of *ICAM1* mRNA in 175 controls, 166 vitiligo patients, 122 generalized vitiligo patients and 44 localized vitiligo patients using Mann-Whitney Wilcoxon test. **(B)** Fold expression of *ICAM1* mRNA in 166 vitiligo patients and 175 controls, as determined by 2^−ΔΔCp^ method. **(C)** Expression of *ICAM1* mRNA with respect to activity of the disease in 121 active vitiligo and 45 stable vitiligo patients using Mann-Whitney Wilcoxon test. **(D)** Expression of *ICAM1* mRNA with respect to gender differences in 87 male and 79 female vitiligo patients using Mann-Whitney Wilcoxon test. **(E)** Expression of *ICAM1* mRNA with respect to different age of onset groups in 166 vitiligo patients using Mann-Whitney Wilcoxon test. **(F)** Expression of *ICAM1* mRNA with respect to *TNFB* +252 A/G in 166 vitiligo patients and 175 controls using Mann-Whitney Wilcoxon test.

Comparison of the findings showed significant increase in expression of *ICAM1* mRNA in 166 vitiligo patients than in 175 unaffected controls after normalization with *GAPDH* expression as suggested by mean ΔCp values (*p* = 0.008) (Fig. 2A). GV patients showed significantly higher mRNA levels of *ICAM1* as compared to LV patients (*p* = 0.002) (Fig. 2A). The 2^−ΔΔCp^ analysis showed approximately 2.7 fold higher expression of *ICAM1* mRNA in patients as compared to controls (Fig. 2B).

In addition, the effect of *ICAM1* expression on progression of the disease i.e. active and stable cases (Fig. 2C) revealed that active vitiligo patients had significantly increased expression of *ICAM1* mRNA as compared to patients with stable vitiligo (*p* = 0.008) suggesting the involvement *ICAM1* in disease progression. Moreover, the susceptibility of the disease was also checked based on the gender differences and we found that female patients with vitiligo showed significantly higher *ICAM1* expression as compared to male patients (*p* = 0.006) (Fig. 2D). When *ICAM1* expression was monitored in different age of onset groups of patients, patients with the age of onset group 1–20 yrs showed significantly increased expression of *ICAM1* mRNA as compared to the age groups 21–40, 41–60 and 61–80 yrs (*p* = 0.009, *p* = 0.004 and *p* = 0.029 respectively) suggesting the importance of *ICAM1* in early onset of the disease (Fig. 2E).

### Correlation of *ICAM1* transcripts with the *TNFB* +252 A/G and exon 3 C/A genotypes

Furthermore, the effect of *TNFB* +252 A/G genotypes was also checked on expression of *ICAM1* mRNA (Fig. 2F). Inevitably, *ICAM1* expression was significantly increased in patients with susceptible GG genotypes as compared to controls (*p* = 0.005). Also, patients with genotypes AG showed significant increase in *ICAM1* mRNA as compared to controls (*p* = 0.001); however, no significant difference was observed in the *ICAM1* expression in patients as compared to controls with AA genotypes (*p* = 0.074) (Fig. 2F). As +252 A/G SNP is strongly linked with the exon 3 C/A SNP, similar results were obtained for *ICAM1* expression with respect to exon 3 C/A genotypes (Fig. S4B in File S2).

## Discussion

TNF plays a central role in the so-called “cytokine storm” characteristic of several autoimmune diseases. As many aspects of the cellular and humoral immune responses are under genetic control and account for individual differences in immune response patterns, there is increasing interest in genetic polymorphisms that affect inflammatory cytokines, which might explain the well-known diversity among clinical findings and outcomes in patients [26]. Therefore, genes encoding cytokines could be considered as the candidate genes for vitiligo susceptibility.

Vitiligo cannot be explained by simple Mendelian genetics, however it is characterized by incomplete penetrance, multiple susceptibility loci and genetic heterogeneity [27]. Autoimmunity has been suggested to play a major role in the pathogenesis of vitiligo since it shows frequent association with other autoimmune disorderse [6], [7]. Furthermore, these autoimmune diseases also occur at elevated frequency among close relatives of vitiligo patients, suggesting that the increased risk of vitiligo and other autoimmune disorders in these families has a genetic basis. Our previous results showed that 21.93% of Gujarat vitiligo patients exhibit positive family history and 13.68% patients have at least one first-degree relative affected [28]. The present study also shows that 12.98% of vitiligo patients have one or more first degree relative affected, suggesting the involvement of genetic factors in vitiligo pathogenesis.

The autoimmune destruction of melanocytes can be explained by the abnormalities in both humoral and cell-mediated immunity [29], [30]. Melanocyte specific circulating autoantibodies [31]–[33], autoreactive CD8^+^ cytotoxic T-cells and macrophages [34], [35] that recognize pigment cell antigens have been detected in the sera of a significant proportion of vitiligo patients. In particular, active cases of vitiligo were demonstrated to have higher levels of autoantibodies and cytotoxic T-cells [36]. Our recent study has also demonstrated circulating autoantibodies in the sera of 75% of Gujarat vitiligo patients as compared to unaffected individuals (*Unpublished data*). The autoimmune hypothesis gains further support from immunotherapy studies of melanoma patients [37]. Twenty six percent of melanoma patients responded to IL2 based immunotherapy, developed vitiligo suggesting that antimelanotic T-cells which might be responsible for melanoma regression may also be linked to the destruction of normal melanocytes in vitiligo [38].

The ultimate pathway of destruction of melanocytes in vitiligo is not known. Apoptosis is one of the cell death pathways suggested for melanocyte destruction. Cytokines such as IFNG, TNFA, TNFB and IL1 released by lymphocytes and keratinocytes can initiate apoptosis [22]. Recently, we have shown increased TNFA protein and transcript levels in vitiligo patients, suggesting an early apoptosis of melanocytes [18]. Moreover, imbalance of cytokines in the epidermal microenvironment of lesional vitiligo skin has been demonstrated, which could impair the life span and function of melanocytes [11], [12], [39], [40].

*TNFA* and *TNFB* polymorphisms are reported to be associated with the inflammatory and immunomodulatory responses and are involved in the modulation of gene expression, and thus affect the precipitation and progression of the autoimmune diseases [19], [21], [41]–[43]. The +252 A/G polymorphism of *TNFB* is present in intron 1 and linked to an amino acid substitution at position 26 of the TNFB protein, the substitution being conserved as asparagine in the *TNFB* +252 G and as threonine in the *TNFB* +252 A allele [19]. The *TNFB* +252 G allele has been shown to be associated with increased expression and the *TNFB* +252 A allele with decreased production of the TNFB protein [19]. The *TNFA* -308 A and the *TNFB* +252 G alleles are constituents of one of the extended ancestral haplotypes (AH8.1) located in the chromosomal region 6p21.3–21.1 (MHC). This haplotype is known to be associated with serious disorders of the immune system [44]–[46]. The *TNFB* +252 G allele was also associated with several autoimmune diseases such as type 1 diabetes mellitus [47], [48] and systemic lupus erythematosus [49], [50]. The present study for the first time reports significant association of *TNFB* +252 A/G polymorphism with vitiligo susceptibility. In particular, we found that +252 G allele was prevalent in GV patients as compared to LV patients and healthy controls. We also found that +252 G allele was prevalent in active cases of vitiligo as compared to patients with stable vitiligo and controls suggesting the involvement of the allele in the progression of the disease. Moreover, +252 G allele was significantly associated with female patients as compared to male patients suggesting that females are more prone to autoimmune diseases.

The whole *TNFB* gene is in strong linkage disequilibrium (LD), therefore the +252 G allele naturally coexists with the 804 A allele of the exon 3 C/A polymorphism (Thr26Asn) [51]. We also found that the two SNPs studied are in perfect LD in our population and provide the same genetic information for the associations, and so as the haplotype. Ozaki *et al.* [21] investigated the functionality of the +252 A/G and 804 C/A SNPs in the *TNFB* gene. The 804 C/A polymorphism causes an amino-acid change from threonine (T) to asparagine (N) at codon 26. They found that the variant protein 26 N is associated with a two fold increase in the induction of cell adhesion molecules in vascular smooth muscle cells [21].

The *TNFB* +252 A/G and exon 3 C/A SNPs could affect susceptibility to vitiligo through its influence on the production of TNFA and TNFB. As *TNFA* and *TNFB* genes are tandemly arranged within the HLA complex, and it has been shown that the *TNFB* gene polymorphisms influence the level of production of the TNFA protein [19], [52]. Studies indicate that variations at both *TNFA* -308 G/A and *TNFB* +252 A/G can affect TNFA production levels [21], [53]. The exon 3 C/A SNP is in strong LD with +252 A/G SNP of intron 1 and the combination of these allelic forms may lead to different levels of TNFA production in response to various physiological and pathological stimuli [54]. This results in variation in predisposition to vitiligo and is also involved in different clinical aspects of the disease. Japanese and Danish studies showed the polymorphism in the *TNFB* gene (exon 3 C/A) to be associated with increased susceptibility to type 2 diabetes mellitus (T2DM) [55], [56].

Pociot *et al*. [57] reported that TNFA levels were highest in *TNFB* +252GG homozygotes, lowest in the *TNFB* +252AA homozygotes, and intermediate in the *TNFB* +252AG heterozygous individuals. *In vitro*, direct analysis of skin T-cells from the margins of vitiliginous skin showed that polarized type-1 T cells (CD4^+^ and particularly CD8^+^), which predominantly secrete IFNG and TNFA are associated with the destruction of melanocytes during active vitiligo [58]. In vitiligo affected skin, a significantly higher expression of *TNFA*, *IL6*, and *IFNG* was detected compared with healthy controls and perilesional, non-lesional skin [11], [12], [39] indicating that cytokine imbalance plays an important role in the depigmentation process of vitiligo. Since elevated levels of TNFA have been associated with vitiligo, this could explain the association of *TNFB* +252 G allele with susceptibility to vitiligo in the present study. Homozygosity for the *TNFB* +252 A allele will be protective because of its association with lower TNFA and TNFB levels. Previously, Hasegawa et al. [59] also suggested the correlation of *TNFB* +252 A/G polymorphism with elevated levels of TNFA in pulmonary fibrosis in scleroderma. *TNFB* +252GG homozygosity has also been shown to be associated with susceptibility to systemic lupus erythematosus in some populations from Germany, Korea, and China [60]–[63].

There are no reports available for *TNFB* gene expression in vitiligo patients till date. Hence, the present study also focuses on the *TNFB* transcript levels in vitiligo patients. Vitiligo patients showed significant increase in *TNFB* transcript levels as compared to controls suggesting that melanocyte death in patients could be triggered due to the increased TNFB levels. For the first time we report that GV has significantly higher *TNFB* transcript levels as compared to LV patients which indicate involvement of autoimmunity in precipitation of GV. Our results also indicate that active vitiligo patients had significantly higher *TNFB* transcript levels as compared to patients with stable vitiligo, which signifies the role of *TNFB* in disease progression. Our results also suggest that there are significantly higher transcript levels of *TNFB* in female patients as compared to male patients suggesting that females have increased susceptibility towards vitiligo as compared to males, implicating gender biasness in the development of autoimmunity [64]–[65]. Furthermore, the genotype-phenotype analysis for +252 A/G polymorphism indicated that patients with GG and AG genotypes had higher expression of *TNFB* transcripts suggesting the crucial role of +252 G allele in pathogenesis of vitiligo. Also, the genotype-phenotype analysis of *TNFB* exon 3 C/A showed that patients harboring AA and CA genotypes had higher expression of *TNFB* suggesting that allele ‘A’ plays an important role in increased expression of *TNFB.* Hence, genetic association studies with *TNFB* polymorphisms in different ethnic populations need to be explored.

TNFA and TNFB also have the capacity to inhibit melanogenesis through an inhibitory effect on tyrosinase and tyrosinase related protein [66]. *In vitro* direct analysis of vitiliginous skin margins showed the presence of polarized CD4^+^ and CD8^+^ T-cells, which predominantly secrete IFNG, TNFA and TNFB, might be associated with the destruction of melanocytes during active disease [67]. Our recent study has also shown dramatic increase in CD8^+^ T-cell number and significant decrease in Tregs number in circulation of active vitiligo patients [68].

Furthermore, IFNG, TNFA and TNFB stimulate the expression of ICAM1 on the cell-surface of melanocytes [23], which is important for activating T-cells and recruiting leukocytes [69], [70]. ICAM1 protein levels are upregulated in vitiligo skin and in melanocytes from perilesional vitiligo skin [71], [24]. The present study also showed increased expression of *ICAM1* in vitiligo patients suggesting that increased *TNFB* levels might be responsible for increased *ICAM1* expression in vitiligo patients and our results are in concordance with the previous reports [24], [70], [72], [73]. In addition, we found that *ICAM1* expression was increased with the susceptible *TNFB +*252GG and exon 3 AA genotypes in patients suggesting the crucial role of *TNFB* and its SNPs in altered *ICAM1* expression. It has been reported that increased expression of ICAM1 on the melanocytes enhances T cell -melanocyte attachment in the skin and may lead to the destruction of melanocytes in vitiligo [24], [71]. Moreover, the *ICAM1* expression was increased in active cases of vitiligo as compared to stable vitiligo suggesting its role in the progression of the disease. The *ICAM1* expression was increased with early age of onset of the disease further implicating the important role of ICAM1 in the early phase of the disease. Also, female patients showed an increased expression of *ICAM1* as compared to male patients suggesting that females have more susceptibility towards vitiligo.

Recently, we have shown positive association of HLA-A*33:01, HLA-B*44:03, and HLA-DRB1*07:01 with vitiligo patients from North India and Gujarat suggesting an autoimmune link of vitiligo in these cohorts [74]. We also showed that the three most significant class II region SNPs: rs3096691 (just upstream of *NOTCH4*), rs3129859 (just upstream of *HLA-DRA*), and rs482044 (between *HLA-DRB1* and *HLA-DQA1*) are associated with GV [75]. The genotype-phenotype correlation of *TNFA, CTLA4*, *IL4*, *MYG1*, *IFNG* and *NALP1* gene polymorphisms supported the autoimmune pathogenesis of vitiligo in Gujarat population [18], [76]–[80]; whereas our earlier studies on *CAT*, *MBL2*, *ACE*, *PTPN22* polymorphisms did not show significant association [81]–[84]. Recently, we have shown that *SOD2* and *SOD3* polymorphisms may be genetic risk factors for susceptibility and progression of vitiligo and contribute to increased transcript levels and activity of SOD2 and SOD3 in patients [85]. It is worth mentioning that TNFB is one of the major transcriptional activator of *SOD2* and *SOD3* genes in different tissues and cell types [86], [87]. Hence, increased *TNFB* production might be responsible for the increased *SOD2* and *SOD3* transcripts [85] which may lead to accumulation of H_2_O_2_ in mitochondrial and extracellular compartments resulting in oxidative damage to the melanocytes.

In conclusion, our findings suggest that the increased *TNFB* levels in vitiligo patients could result, at least in part, from variations at the genetic level. Moreover, the increased *TNFB* levels in patients may lead to increased *ICAM1* expression which is probably an important link between cytokines and T cells involved in vitiligo pathogenesis. For the first time, we show that the +252 A/G and exon 3 C/A polymorphisms of the *TNFB* gene are associated with vitiligo and influence the *TNFB* expression levels. The study also emphasizes the influence of *TNFB* on the disease progression, onset of the disease and gender biasness for developing vitiligo. More detailed studies regarding the role of *TNFB* in precipitation of vitiligo and the development of effective anti-TNF agents may prove to be useful as preventive/ameliorative therapies.

## Supporting Information

File S1 **Supplementary Tables. Table S1.** Primers and restriction enzymes used for *TNFB* +252G/A SNP genotyping and gene expression analyses. **Table S2.** Association studies for *TNFB* gene +252A/G polymorphism in male and female vitiligo patients from Gujarat. **Table S3.** Association studies for *TNFB* gene exon 3 C/A polymorphism in vitiligo patients and controls from Gujarat. **Table S4.** Association studies for *TNFB* gene exon 3 C/A polymorphism in different clinical types of vitiligo patients and controls from Gujarat.
(DOC)

File S2 **Supplementary Figures.** **Figure S1.** PCR-RFLP analysis of *TNFB* +252A/G polymorphism on 2.0 % agarose gel electrophoresis: lanes: 1, 3, 4, 5 & 7 show homozygous (GG) genotypes; lane: 2 shows homozygous (AA) genotype; lane: 6 shows heterozygous (AG) genotype. **Figure S2. TaqMan end point fluoroscence analysis for** ***TNFB*** **C/A; (Thr26Asn) polymorphism** using dual color hydrolysis probes (FAM and VIC) by LightCycler®480Real-Time PCR protocol. The three genotypes identified as: AA, AC and CC, based on fluorescence with Channel 465-510 (FAM for ‘A’ allele) and Channel 536-580 (VIC for ‘C’ allele). A no-template control (NTC) was used with each SNP genotyping assay (shown as grey spot). **Figure S3. Melt curve analysis of** ***TNFB, ICAM1*** **and** ***GAPDH*** **showing specific amplification. Figure S4. Relative gene expression of** ***TNFB*** **and** ***ICAM1*** **with respect to** ***TNFB*** **exon 3 C/A in controls and vitiligo patients: (A)** Expression of *TNFB* mRNA with respect to *TNFB* exon 3 C/A in 166 vitiligo patients and 175 controls using Mann-Whitney Wilcoxon test [MeanΔCp ± SEM: Controls CC vs Patients CC: 8.102 ± 0.1747 vs 7.678 ± 0.2379 (*p* = 0.168); Controls CA vs Patients CA; 7.761 ± 0.0952 vs 7.028 ± 0.3376 (p = 0.039); Controls AA vs Patients AA: 6.918 ± 0.2808 vs 5.217 ± 0.5087 (*p* = 0.015) (*NS*  =  non-significant)].** (B)** Expression of *ICAM1* mRNA with respect to *TNFB* exon 3 C/A in 166 vitiligo patients and 175 controls using Mann-Whitney Wilcoxon test [MeanΔCp ± SEM; Controls CC vs Patients CC: 8.767 ± 0.2267 vs 8.133 ± 0.2658 (*p* = 0.074); Controls CA vs Patients CA: 8.494 ± 0.2434 vs 7.225 ± 0.2729 (*p* = 0.001); Controls AA vs Patients AA: 8.007 ± 0.3297 vs 6.327 ± 0.4397 (*p* = 0.005).
(DOCX)
