# Supplementary material for: Tumor Necrosis Factor B (TNFB) Genetic Variants and Its Increased Expression Are Associated with Vitiligo Susceptibility
Source: PLoS One. 2013 Nov 27;8(11):e81736. doi: 10.1371/journal.pone.0081736 (PMC3842287; doi:10.1371/journal.pone.0081736)
Supplement: File S1 — Supplementary Tables. Table S1. Primers and restriction enzymes used for TNFB +252G/A SNP genotyping and gene expression analyses. Table S2. Association studies for TNFB gene +252A/G polymorphism in male and female vitiligo patients from Gujarat. Table S3. Association studies for TNFB gene exon 3 C/A polymorphism in vitiligo patients and controls from Gujarat. Table S4. Association studies for TNFB gene exon 3 C/A polymorphism in different clinical types of vitiligo patients and controls from Gujarat. (DOC) [file pone.0081736.s001.doc]

**File S1: Supplementary Tables:**

**Table S1.** Primers and restriction enzymes used for *TNFB* +252G/A SNP genotyping and gene expression analyses.

| **Gene/SNP** | **Primer Sequence (5’ to 3’)** | **Annealing**  **Temperature**  **(°C)** | **Amplicon size**  **(bp)** | **Restriction Enzyme (Digested Products)** |
| --- | --- | --- | --- | --- |
| **(rs909253)**  *TNFB*  +252G/A F  *TNFB*  +252G/A R  *TNFB*  expression F  *TNFB*  expression R  *ICAM1*  expression F  *ICAM1*  expression R  *GAPDH* expression F  *GAPDH* expression R | GGTGGTGTCATGGGGAGAACC  GGGCCTTGGTGGGTTTGGTT  GGGCCTTGGTTCTCCCCATG  CTGGGGTCTCCAATGAGGTGA  TTGGAAAGAGGAGAGTGACAG  GGACATTCAAGTCAGTTACCGA  ATCCCATCACCATCTTCCAGGA  CAAATGAGCCCCAGCCTTCT | 62  65  65  65 | 417    232    212  122 | *Nco*I  (284 &  137 bp)  -  -  - |
|  |  |  |  |

**Table S2**. Association studies for *TNFB* gene +252A/G polymorphism in male and female vitiligo patients from Gujarat.

| **SNP** | **Genotype or allele** | **Male Patients**  **(Freq.)** | **Female Patients**  **(Freq.)** | ***p* for Association** | ***p* for HWE** | **Odds ratio**  **(95% CI)** |
| --- | --- | --- | --- | --- | --- | --- |
| rs909253  (+252A/G) | Genotype | n = 224 | n = 300 | 0.039^a^  0.021^b^ | 0.909  (M)  0.054  (F) | 0.714 (0.540-0.945) |
|  | AA  AG  GG  Allele  A  G | 131(0.58)  81 (0.37)  12 (0.05)  343 (0.77)  105 (0.23) | 154 (0.51)  112 (0.38)  34 (0.11)  420 (0.70)  180 (0.30) |  |  |  |

‘n’ represents number of male and female vitiligo patients, HWE refers to Hardy-Weinberg Equilibrium, CI refers to confidence interval, (M) refers to male patients and (F) refers to female patients,

^a^ male patients vs. female patients using chi-square test with 3 × 2 contingency table,

^b^ male patients vs. female patients using chi-square test with 2 × 2 contingency table,

values are significant at *p* ≤ 0.025 due to Bonferroni’s correction.

**Table S3**. Association studies for *TNFB* gene exon 3 C/A polymorphism in vitiligo patients and controls from Gujarat.

| **SNP** | **Genotype or allele** | **Vitiligo Patients**  **(Freq.)** | **Controls**  **(Freq.)** | ***p* for Association** | ***p* for HWE** | **Odds ratio**  **(95% CI)** |
| --- | --- | --- | --- | --- | --- | --- |
| rs1041981 (exon 3 C/A; Thr26Asn) | Genotype | n = 524 | n = 592 | 0.002^a^  0.001^b^ | 0.109  (Patients)  0.389  (Controls) | 1.424 (1.171-1.732) |
|  | CC  CA  AA  Allele  C  A | 285 0.54)  193 (0.37)  46 (0.09)  763 (0.73)  285 (0.27) | 375 (0.63)  188 (0.32)  29 (0.05)  938 (0.79)  246 (0.21) |  |  |  |
| rs1041981 (exon 3 C/A; Thr26Asn) | Genotype | **Male Patients**  **(Freq.)**  n= 224 | **Female Patients**  **(Freq.)**  n= 300 | 0.039^c^  0.021^d^ | 0.909  (M)  0.054  (F) | 0.714 (0.540-0.945) |
|  | CC  CA  AA  Allele  C  A | 131(0.58)  81 (0.37)  12 (0.05)  343 (0.77)  105 (0.23) | 154 (0.51)  112 (0.38)  34 (0.11)  420 (0.70)  180 (0.30) |  |  |  |

‘n’ represents number of patients/ controls, HWE refers to Hardy-Weinberg Equilibrium, CI refers to confidence interval, (M) refers to male patients and (F) refers to female patients,

^a^ vitiligo patients vs. controls using chi-square test with 3 × 2 contingency table,

^b^ vitiligo patients vs. controls using chi-square test with 2 × 2 contingency table,

^c^ male patients vs. female patients using chi-square test with 3×2 contingency table,

^d^ male patients vs. female patients using chi-square test with 2×2 contingency table,

values are significant at *p* ≤ 0.025 due to Bonferroni’s correction.

**Table S4**. Association studies for *TNFB* gene exon 3 C/A polymorphism in different clinical types of vitiligo patients and controls from Gujarat.

| **SNP** | **Genotype or allele** | **Generalized Vitiligo Patients**  **(Freq.)** | **Localized**  **Vitiligo Patients**  **(Freq.)** | **Controls**  **(Freq.)** | ***p* for Association** | ***p* for HWE** | **Odds ratio**  **(95% CI)** |
| --- | --- | --- | --- | --- | --- | --- | --- |
| rs1041981 (exon 3 C/A; Thr26Asn) | Genotype | n = 360 | n = 164 | n = 592 | 0.030^a^  <0.0001^b^  0.826^c^  0.006^a^  <0.0001^b^  0.759^c^ | 0.083  (GV)  0.286  (LV)  0.389  (Controls) | 1.541^a^ (1.133-2.096)  1.623^b^  (1.312-2.008)  1.053^c^ (0.782-1.419) |
|  | CC  CA  AA  Allele  C  A | 184 (0.51)  137 (0.38)  39 (0.11)  505 (0.70)  215 (0.30) | 103 (0.63)  51 (0.31)  10 (0.06)    257 (0.78)  71 (0.22) | 375(0.63)  188(0.32)  29 (0.05)  938(0.79)  246(0.21) |  |  |  |
| rs1041981 (exon 3 C/A; Thr26Asn) | Genotype | **Active**  **Vitiligo**  **(Freq.)**  n = 393 | **Stable**  **Vitiligo**  **(Freq.)**  n = 131 | **Controls**  **(Freq.)**  n = 592 | 0.012^d^  < 0.0001^e^  0.945^f^  0.002 ^d^  <0.0001^e^  0.800^f^ | 0.163  (AV)  0.645  (SV)  0.389  (Controls) | 1.691^d^ (1.204-2.376)  1.597 ^e^ (1.297-1.966)  0.944 ^f^  (0.676-  1.319) |
|  | CC  CA  AA  Allele  C  A | 201 (0.51)  152 (0.39)  40 (0.10)  554 (0.70)  232 (0.30) | 85 (0.65)  40 (0.30)  6 (0.05)  210 (0.80)  52 (0.20) | 375 (0.63)  188 (0.32)  29 (0.05)  938(0.79)  246(0.21) |  |  |  |

‘n’ represents number of patients/ controls, HWE refers to Hardy-Weinberg Equilibrium, CI refers to confidence interval, GV refers to generalized vitiligo, LV refers to localized vitiligo, AV refers to active vitiligo, SV refers to stable vitiligo,

^a^ generalized vitiligo vs. localized vitiligo, ^b^ generalized vitiligo vs. controls,

^c^ localized vitiligo vs. controls, ^d^ active vitiligo vs. stable vitiligo,

^e^ active vitiligo vs. controls, ^f^ stable vitiligo vs. controls,

values are significant at *p* ≤ 0.025 due to Bonferroni’s correction.
