# Supplementary material for: Tumor Necrosis Factor B (TNFB) Genetic Variants and Its Increased Expression Are Associated with Vitiligo Susceptibility
Source: PLoS One. 2013 Nov 27;8(11):e81736. doi: 10.1371/journal.pone.0081736 (PMC3842287; doi:10.1371/journal.pone.0081736)

**File S2: Supplementary Figures**

**Figure S1. PCR-RFLP analysis of *TNFB* +252A/G polymorphism on 2.0 % agarose gel electrophoresis:** lanes: 1, 3, 4, 5 & 7 show homozygous (GG) genotypes; lane: 2 shows homozygous (AA) genotype; lane: 6 shows heterozygous (AG) genotype.

**Figure S2. TaqMan end point fluoroscence analysis for *TNFB* C/A; (Thr26Asn) polymorphism** using dual color hydrolysis probes (FAM and VIC) by LightCycler®480Real-Time PCR protocol. The three genotypes identified as: AA, AC and CC, based on fluorescence with Channel 465-510 (FAM for ‘A’ allele) and Channel 536-580 (VIC for ‘C’ allele). A no-template control (NTC) was used with each SNP genotyping assay (shown as grey spot).

**Figure S3. Melt curve analysis of *TNFB*, *ICAM1* and *GAPDH* showing specific amplification.**

**Figure S4. Relative gene expression of *TNFB* and *ICAM1* with respect to *TNFB* exon 3 C/A in controls and vitiligo patients:**

**(A)** Expression of *TNFB* mRNA with respect to *TNFB* exon 3 C/A in 166 vitiligo patients and 175 controls using Mann-Whitney Wilcoxon test [Mean∆Cp ± SEM: Controls CC vs Patients CC: 8.102 ± 0.1747 vs 7.678 ± 0.2379 (*p=*0.168); Controls CA vs Patients CA: 7.761 ± 0.0952 vs 7.028 ± 0.3376 (*p=*0.039); Controls AA vs Patients AA: 6.918 ± 0.2808 vs 5.217 ± 0.5087 (*p=*0.015) (*NS* = non-significant)].

**(B)** Expression of *ICAM1* mRNA with respect to *TNFB* exon 3 C/A in 166 vitiligo patients and 175 controls using Mann-Whitney Wilcoxon test [Mean∆Cp ± SEM: Controls CC vs Patients CC: 8.767 ± 0.2267 vs 8.133 ± 0.2658 (*p=*0.074); Controls CA vs Patients CA: 8.494 ± 0.2434 vs 7.225 ± 0.2729 (*p=*0.001); Controls AA vs Patients AA: 8.007 ± 0.3297 vs 6.327 ± 0.4397 (*p=*0.005).


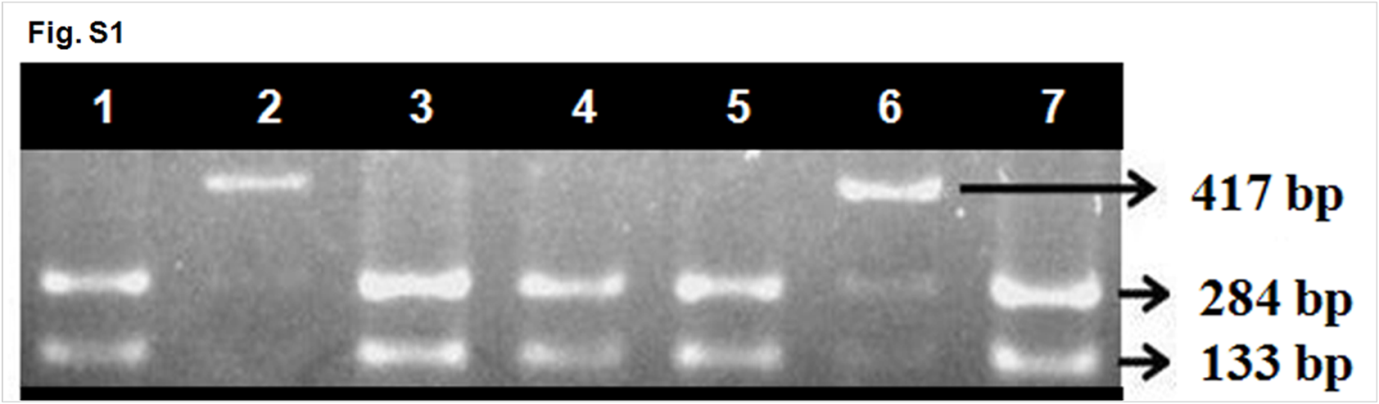


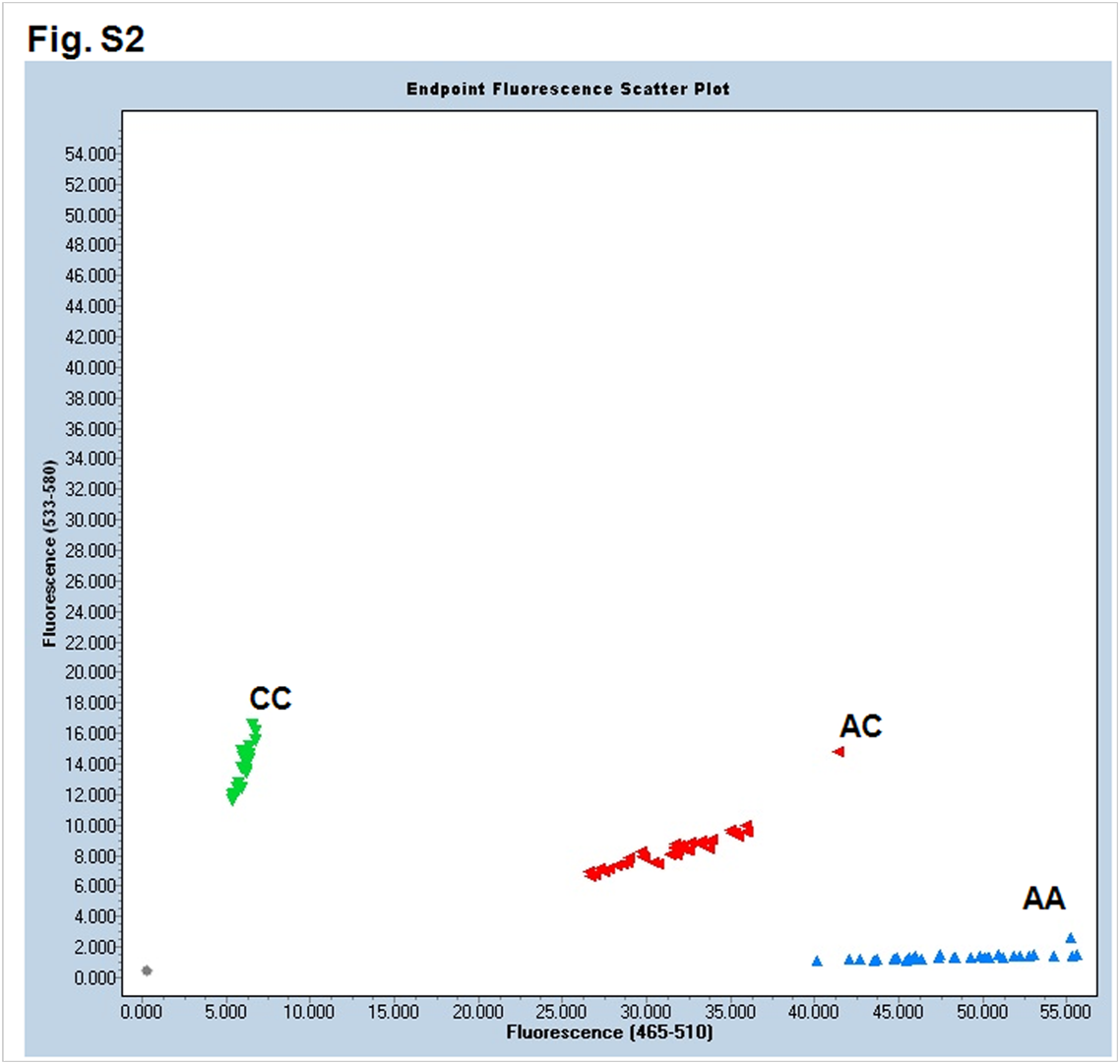


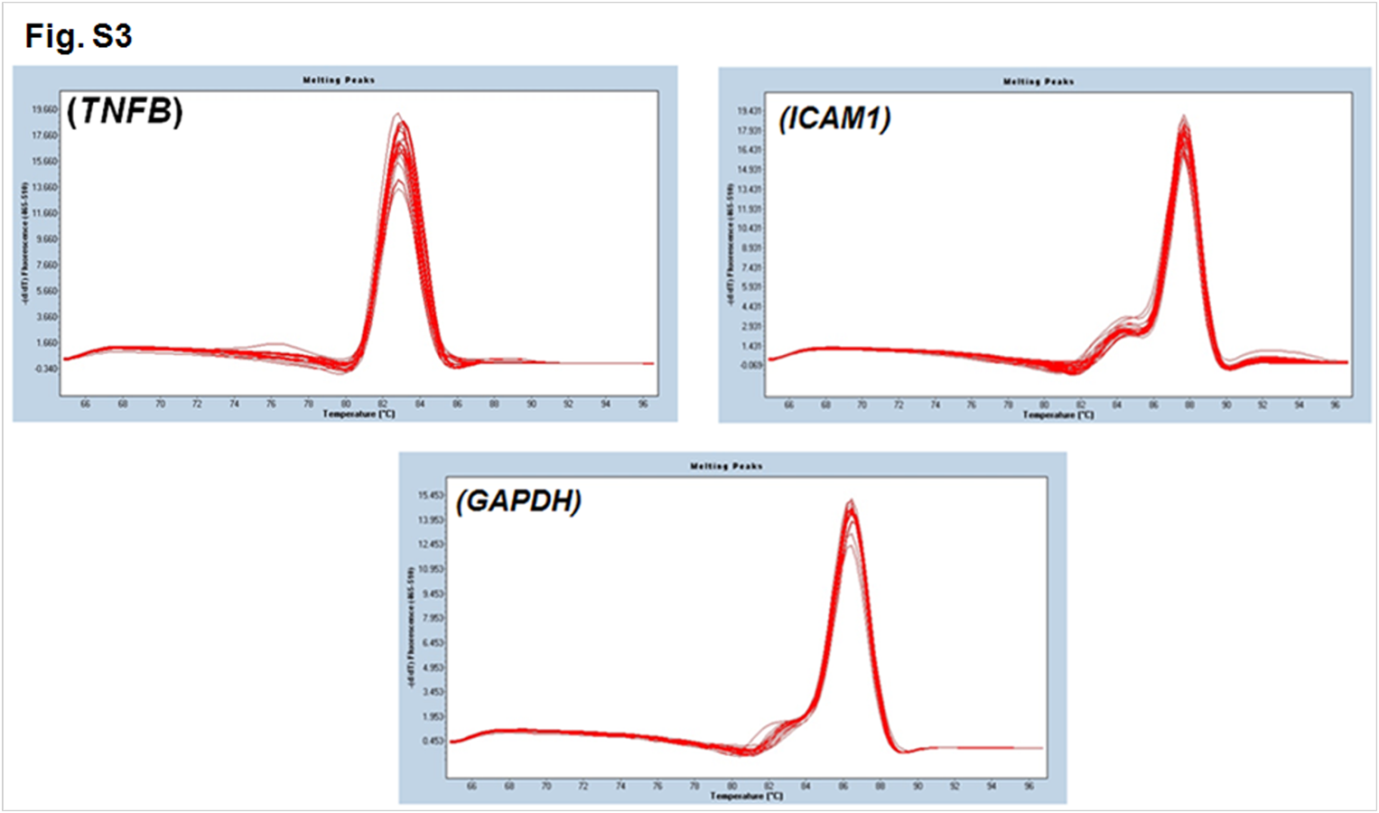


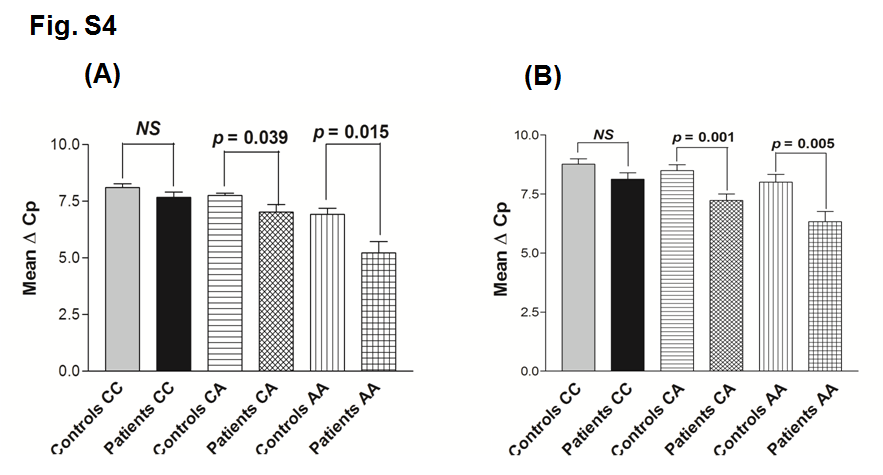

Supplement: File S2 — Supplementary Figures. Figure S1. PCR-RFLP analysis of TNFB +252A/G polymorphism on 2.0 % agarose gel electrophoresis: lanes: 1, 3, 4, 5 & 7 show homozygous (GG) genotypes; lane: 2 shows homozygous (AA) genotype; lane: 6 shows heterozygous (AG) genotype. Figure S2. TaqMan end point fluoroscence analysis for TNFB C/A; (Thr26Asn) polymorphism using dual color hydrolysis probes (FAM and VIC) by LightCycler®480Real-Time PCR protocol. The three genotypes identified as: AA, AC and CC, based on fluorescence with Channel 465-510 (FAM for ‘A’ allele) and Channel 536-580 (VIC for ‘C’ allele). A no-template control (NTC) was used with each SNP genotyping assay (shown as grey spot). Figure S3. Melt curve analysis of TNFB, ICAM1 and GAPDH showing specific amplification. Figure S4. Relative gene expression of TNFB and ICAM1 with respect to TNFB exon 3 C/A in controls and vitiligo patients: (A) Expression of TNFB mRNA with respect to TNFB exon 3 C/A in 166 vitiligo patients and 175 controls using Mann-Whitney Wilcoxon test [MeanΔCp ± SEM: Controls CC vs Patients CC: 8.102 ± 0.1747 vs 7.678 ± 0.2379 (p = 0.168); Controls CA vs Patients CA; 7.761 ± 0.0952 vs 7.028 ± 0.3376 (p = 0.039); Controls AA vs Patients AA: 6.918 ± 0.2808 vs 5.217 ± 0.5087 (p = 0.015) (NS = non-significant)]. (B) Expression of ICAM1 mRNA with respect to TNFB exon 3 C/A in 166 vitiligo patients and 175 controls using Mann-Whitney Wilcoxon test [MeanΔCp ± SEM; Controls CC vs Patients CC: 8.767 ± 0.2267 vs 8.133 ± 0.2658 (p = 0.074); Controls CA vs Patients CA: 8.494 ± 0.2434 vs 7.225 ± 0.2729 (p = 0.001); Controls AA vs Patients AA: 8.007 ± 0.3297 vs 6.327 ± 0.4397 (p = 0.005). (DOCX) [file pone.0081736.s002.docx]
